# Supplementary material for: A decade of ‘blue tourism’ sustainability research: Exploring the impact of cruise tourism on coastal areas
Source: Camb Prism Coast Futur. 2023 Jan 19;1:e12. doi: 10.1017/cft.2023.2 (PMC12337236; doi:10.1017/cft.2023.2)
Supplement: Supplementary file 1 [file S2754720523000021sup001.docx]

**APPEDIX A: Bibliographic Dataset**

Cruise Tourism Review and Research-Articles published between 2012 and 2022

1. Ajagunna, I., & Casanova, S. (2022). An analysis of the post-COVID-19 cruise industry: Could this be a new possibility for the luxury yacht sector in the Caribbean? Worldwide Hospitality and Tourism Themes, 14(2), 115–123. https://doi.org/10.1108/WHATT-12-2021-0158
2. Ajagunna, I., & Crick, A. P. (2014). Managing interactions in the tourism industry – a strategic tool for success. Worldwide Hospitality and Tourism Themes, 6(2), 179–190. https://doi.org/10.1108/WHATT-12-2013-0051
3. Ajagunna, I., Ilori, M. O., & McLean, E. (2022a). An analysis of post-pandemic scenarios: What are the prospects for the Caribbean cruise industry? Worldwide Hospitality and Tourism Themes, 14(2), 91–98. https://doi.org/10.1108/WHATT-12-2021-0159
4. Ajagunna, I., Ilori, M. O., & McLean, E. (2022b). What are the implications for the post-pandemic Caribbean cruise industry? Worldwide Hospitality and Tourism Themes, 14(2), 193–198. https://doi.org/10.1108/WHATT-12-2021-0161
5. Alberini, C. (2021). A holistic approach towards a more sustainable urban and port planning in tourist cities. International Journal of Tourism Cities, 7(4), 1076–1089. https://doi.org/10.1108/IJTC-02-2021-0028
6. Amaya-Vías, D., Nebot, E., & López-Ramírez, J. A. (2018). Comparative studies of different membrane distillation configurations and membranes for potential use on board cruise vessels. Desalination, 429, 44–51. https://doi.org/10.1016/j.desal.2017.12.008
7. Andrade, A., & Smith, K. A. (2020). Tourism distribution in small island destinations: The case of Fernando de Noronha, Brazil. Journal of Hospitality and Tourism Insights, 3(2), 171–189. https://doi.org/10.1108/JHTI-04-2019-0060
8. Ardoin, N. M., Wheaton, M., Hunt, C. A., Schuh, J. S., & Durham, W. H. (2016). Post-trip philanthropic intentions of nature-based tourists in Galapagos. Journal of Ecotourism, 15(1), 21–35. https://doi.org/10.1080/14724049.2016.1142555
9. Artal-Tur, A., Navarro-Azorín, J. M., & Ramos-Parreño, J. M. (2019). Estimating the impact of cruise tourism through regional input–output tables. Anatolia, 30(2), 235–245. https://doi.org/10.1080/13032917.2018.1519209
10. Bagis, O., & Dooms, M. (2014). Turkey’s potential on becoming a cruise hub for the East Mediterranean Region: The case of Istanbul. Cruises and Cruise Ports: Structures and Strategies, 13, 6–15. https://doi.org/10.1016/j.rtbm.2014.10.008
11. Baños-Pino, J. F., Boto-García, D., Valle, E. D., & Zapico, E. (2022). Is visitors’ expenditure at destination influenced by weather conditions? Current Issues in Tourism, 1–19. https://doi.org/10.1080/13683500.2022.2058468
12. Barbosa, A., Varsani, A., Morandini, V., Grimaldi, W., Vanstreels, R. E. T., Diaz, J. I., Boulinier, T., Dewar, M., González-Acuña, D., Gray, R., McMahon, C. R., Miller, G., Power, M., Gamble, A., & Wille, M. (2021). Risk assessment of SARS-CoV-2 in Antarctic wildlife. Science of The Total Environment, 755, 143352. https://doi.org/10.1016/j.scitotenv.2020.143352
13. Baumann, A. C. (2021). On the path towards understanding overtourism – cruise tourism and the transportation infrastructure. World Leisure Journal, 63(1), 5–13. https://doi.org/10.1080/16078055.2021.1887995
14. Brandajs, F., & Russo, A. P. (2021). Whose is that square? Cruise tourists’ mobilities and negotiation for public space in Barcelona. Applied Mobilities, 6(3), 289–313. https://doi.org/10.1080/23800127.2019.1576257
15. Brida, J. G., Pulina, M., Riaño, E., & Zapata-Aguirre, S. (2012). Cruise passengers’ experience embarking in a Caribbean home port. The case study of Cartagena de Indias. Ocean & Coastal Management, 55, 135–145. https://doi.org/10.1016/j.ocecoaman.2011.10.003
16. Brida, J. G., Pulina, M., Riaño, E., & Zapata-Aguirre, S. (2012). Cruise visitors’ intention to return as land tourists and to recommend a visited destination. Anatolia, 23(3), 395–412. https://doi.org/10.1080/13032917.2012.712873
17. Brida, J. G., Bukstein, D., & Tealde, E. (2015). Exploring cruise ship passenger spending patterns in two Uruguayan ports of call. Current Issues in Tourism, 18(7), 684–700. https://doi.org/10.1080/13683500.2013.861391
18. Chen, C.-A. (2016). How can Taiwan create a niche in Asia’s cruise tourism industry? Tourism Management, 55, 173–183. https://doi.org/10.1016/j.tourman.2016.02.015
19. Cajaiba-Santana, G., Faury, O., & Ramadan, M. (2020). The emerging cruise shipping industry in the arctic: Institutional pressures and institutional voids. Annals of Tourism Research, 80, 102796. https://doi.org/10.1016/j.annals.2019.102796
20. Callejas-Jiménez, M. E., Alcérreca-Huerta, J. C., & Carrillo, L. (2021). Assessment of marine energy-biotopes for Cozumel Island’s reefs: A resource for tourism and renewable ocean energy. Ocean & Coastal Management, 210, 105701. https://doi.org/10.1016/j.ocecoaman.2021.105701
21. Carić, H., & Mackelworth, P. (2014). Cruise tourism environmental impacts – The perspective from the Adriatic Sea. Ocean & Coastal Management, 102, 350–363. https://doi.org/10.1016/j.ocecoaman.2014.09.008
22. Carić, H., Klobučar, G., & Štambuk, A. (2016). Ecotoxicological risk assessment of antifouling emissions in a cruise ship port. Journal of Cleaner Production, 121, 159–168. https://doi.org/10.1016/j.jclepro.2014.08.072
23. Carić, H. (2016). Challenges and prospects of valuation – cruise ship pollution case. Special Volume: Sustainable Tourism: Progress, Challenges and Opportunities, 111, 487–498. https://doi.org/10.1016/j.jclepro.2015.01.033
24. Casado-Díaz, A. B., Navarro-Ruiz, S., Nicolau, J. L., & Ivars-Baidal, J. (2021). Expanding our understanding of cruise visitors’ expenditure at destinations: The role of spatial patterns, onshore visit choice and cruise category. Tourism Management, 83, 104199. https://doi.org/10.1016/j.tourman.2020.104199
25. Castillo-Manzano, J. I., Lopez-Valpuesta, L., & Alanís, F. J. (2015). Tourism managers’ view of the economic impact of cruise traffic: The case of southern Spain. Current Issues in Tourism, 18(7), 701–705. https://doi.org/10.1080/13683500.2014.907776
26. Chen, J. S., Wang, W., Jensen, O., Kim, H., & Liu, W.-Y. (2021). Perceived impacts of tourism in the Arctic. Journal of Tourism and Cultural Change, 19(4), 494–508. https://doi.org/10.1080/14766825.2020.1735403
27. Cheung, W. (Wai Y., Bauer, T., & Deng, J. (2019). The growth of Chinese tourism to Antarctica: A profile of their connectedness to nature, motivations, and perceptions. The Polar Journal, 9(1), 197–213. https://doi.org/10.1080/2154896X.2019.1618552
28. Coggins Jr, A. O. (2014). The globalization of the cruise industry: A tale of ships. Worldwide Hospitality and Tourism Themes, 6(2), 138–151. https://doi.org/10.1108/WHATT-12-2013-0048
29. Colavitti, A. M., & Usai, A. (2019). Applying the HUL approach to walled towns of Mediterranean seaport cities. Journal of Place Management and Development, 12(3), 338–364. https://doi.org/10.1108/JPMD-03-2018-0025
30. Connell, J. (2021). COVID-19 and tourism in Pacific SIDS: lessons from Fiji, Vanuatu and Samoa? The Round Table, 110(1), 149–158. https://doi.org/10.1080/00358533.2021.1875721
31. da Luz, L. M., Antunes, A. P., Caldeirinha, V., Caballé-Valls, J., & Garcia-Alonso, L. (2021). Cruise destination characteristics and performance: Application of a conceptual model to North Atlantic islands of Macaronesia. Research in Transportation Business & Management, 100747. https://doi.org/10.1016/j.rtbm.2021.100747
32. Dawson, J., Stewart, E. J., Johnston, M. E., & Lemieux, C. J. (2016). Identifying and evaluating adaptation strategies for cruise tourism in Arctic Canada. Journal of Sustainable Tourism, 24(10), 1425–1441. https://doi.org/10.1080/09669582.2015.1125358
33. Dawson, J., Johnston, M., & Stewart, E. (2017). The unintended consequences of regulatory complexity: The case of cruise tourism in Arctic Canada. Marine Policy, 76, 71–78. https://doi.org/10.1016/j.marpol.2016.11.002
34. Del Chiappa, G., Lorenzo-Romero, C., & Gallarza, M. (2018). Host community perceptions of cruise tourism in a homeport: A cluster analysis. Journal of Destination Marketing & Management, 7, 170–181. https://doi.org/10.1016/j.jdmm.2016.08.011
35. Dimitrovski, D., Lemmetyinen, A., Nieminen, L., & Pohjola, T. (2021). Understanding coastal and marine tourism sustainability—A multi-stakeholder analysis. Journal of Destination Marketing & Management, 19, 100554. https://doi.org/10.1016/j.jdmm.2021.100554
36. Domènech, A., Gutiérrez, A., & Anton Clavé, S. (2020). Cruise Passengers’ Spatial Behaviour and Expenditure Levels at Destination. Tourism Planning & Development, 17(1), 17–36. https://doi.org/10.1080/21568316.2019.1566169
37. Jordan, E. J., Vieira, J. C., Santos, C. M., & Huang, T.-Y. (Tim). (2020). Do residents differentiate between the impacts of tourism, cruise tourism, and Airbnb tourism? Journal of Sustainable Tourism, 1–19. https://doi.org/10.1080/09669582.2020.1833894
38. Stewart, E. J., Dawson, J., Howell, S. E. L., Johnston, M. E., Pearce, T., & Lemelin, H. (2013). Local-level responses to sea ice change and cruise tourism in Arctic Canada’s Northwest Passage. Polar Geography, 36(1–2), 142–162. https://doi.org/10.1080/1088937X.2012.705352
39. Stewart, E. J., Liggett, D., Lamers, M., Ljubicic, G., Dawson, J., Thoman, R., Haavisto, R., & Carrasco, J. (2020). Characterizing polar mobilities to understand the role of weather, water, ice and climate (WWIC) information. Polar Geography, 43(2–3), 95–119. https://doi.org/10.1080/1088937X.2019.1707319
40. Stewart, E., Dawson, J., & Johnston, M. (2015). Risks and opportunities associated with change in the cruise tourism sector: Community perspectives from Arctic Canada. The Polar Journal, 5(2), 403–427. https://doi.org/10.1080/2154896X.2015.1082283
41. Eikeland, O. F., Apostoleris, H., Santos, S., Ingebrigtsen, K., Boström, T., & Chiesa, M. (2020). Rethinking the role of solar energy under location specific constraints. Energy, 211, 118838. https://doi.org/10.1016/j.energy.2020.118838
42. Fernández-Gámez, M. A., Valcarce-Ruiz, L., Becerra-Vicario, R., & Diéguez-Soto, J. (2022). A dynamic modelling approach to manage the cruise port of call. Research in Transportation Business & Management, 100818. https://doi.org/10.1016/j.rtbm.2022.100818
43. Ferrante, M., De Cantis, S., & Shoval, N. (2018). A general framework for collecting and analysing the tracking data of cruise passengers at the destination. Current Issues in Tourism, 21(12), 1426–1451. https://doi.org/10.1080/13683500.2016.1194813
44. Frame, B., & Hemmings, A. D. (2020). Coronavirus at the end of the world: Antarctica matters. Social Sciences & Humanities Open, 2(1), 100054. https://doi.org/10.1016/j.ssaho.2020.100054
45. Franklyn-Green, L.-G. K., Meredith, N., & Ajagunna, I. (2022). The Caribbean cruise industry and COVID-19: What planned strategic options exist for recovery for small island countries? Worldwide Hospitality and Tourism Themes, 14(2), 162–168. https://doi.org/10.1108/WHATT-12-2021-0151
46. Wang, G., Chang, W.-H., Cui, Y., Qi, G., & Li, K. X. (2020). Introducing an economic impact platform to navigate cruise value-added chain with environmental considerations. Marine Policy, 112, 103713. https://doi.org/10.1016/j.marpol.2019.103713
47. Garay, L. A., Cànoves, G., & Prat, J. M. (2014). Barcelona, a Leader Destination in Cruise-passenger Tourism: Keys, Impacts and Facts. International Journal of Tourism Sciences, 14(1), 23–49. https://doi.org/10.1080/15980634.2014.11434683
48. Gössling, S., Scott, D., & Hall, C. M. (2015). Inter-market variability in CO2 emission-intensities in tourism: Implications for destination marketing and carbon management. Tourism Management, 46, 203–212. https://doi.org/10.1016/j.tourman.2014.06.021
49. Gössling, S., Lohmann, M., Grimm, B., & Scott, D. (2017). Leisure travel distribution patterns of Germans: Insights for climate policy. Case Studies on Transport Policy, 5(4), 596–603. https://doi.org/10.1016/j.cstp.2017.10.001
50. Gould, K. A. (2017). Ecotourism under pressure: The political economy of oil extraction and cruise ship tourism threats to sustainable development in Belize. Environmental Sociology, 3(3), 237–247. https://doi.org/10.1080/23251042.2017.1308238
51. Grythe, H., & Lopez-Aparicio, S. (2021). The who, why and where of Norway’s CO2 emissions from tourist travel. Environmental Advances, 5, 100104. https://doi.org/10.1016/j.envadv.2021.100104
52. Guerra, M., & Dawson, S. M. (2016). Boat-based tourism and bottlenose dolphins in Doubtful Sound, New Zealand: The role of management in decreasing dolphin-boat interactions. Tourism Management, 57, 3–9. https://doi.org/10.1016/j.tourman.2016.05.010
53. Li, H., Zhu, L., & Zheng, M. (2022). Is the cruise enterprise giant also a giant of social responsibility for sustainable development？. Marine Policy, 138, 105009. https://doi.org/10.1016/j.marpol.2022.105009
54. Halter, N. (2020). Tourists fraternising in Fiji in the 1930s. Journal of Tourism History, 12(1), 27–47. https://doi.org/10.1080/1755182X.2019.1682688
55. Han, H., Yu, J., Koo, B., & Kim, W. (2019). Vacationers’ norm-based behavior in developing environmentally sustainable cruise tourism. Journal of Quality Assurance in Hospitality & Tourism, 20(1), 89–106. https://doi.org/10.1080/1528008X.2018.1483287
56. Harkison, T., & Barðadóttir, Þ. (2019). Are we poles apart? Stakeholders’ cooperation and decision-making in on-land cruise tourism in Iceland and New Zealand. Research in Hospitality Management, 9(2), 99–108. https://doi.org/10.1080/22243534.2019.1689699
57. Hillmer-Pegram, K. (2016). Integrating Indigenous values with capitalism through tourism: Alaskan experiences and outstanding issues. Journal of Sustainable Tourism, 24(8–9), 1194–1210. https://doi.org/10.1080/09669582.2016.1182536
58. Ilori, M. O., Ajagunna, I., Oluseyi, P. O., Lawal-Are, A., & Ogundipe, O. T. (2022). Surmounting challenges and breaking barriers: Will the cruise liners open up new frontiers in the new global village? Worldwide Hospitality and Tourism Themes, 14(2), 187–192. https://doi.org/10.1108/WHATT-12-2021-0160
59. Chen, J. M., Petrick, J. F., Papathanassis, A., & Li, X. (2019). A meta-analysis of the direct economic impacts of cruise tourism on port communities. Tourism Management Perspectives, 31, 209–218. https://doi.org/10.1016/j.tmp.2019.05.005
60. Chen, J. M., & Nijkamp, P. (2018). Itinerary planning: Modelling cruise lines’ lengths of stay in ports. International Journal of Hospitality Management, 73, 55–63. https://doi.org/10.1016/j.ijhm.2018.02.005
61. McCarthy, J. (2018a). Responsible cruise tourism and regeneration: The case of Nanaimo, British Columbia, Canada. International Planning Studies, 23(3), 225–238. https://doi.org/10.1080/13563475.2018.1428539
62. Chen, J. S., & Chen, Y.-L. (2016). Tourism stakeholders’ perceptions of service gaps in Arctic destinations: Lessons from Norway’s Finnmark region. Journal of Outdoor Recreation and Tourism, 16, 1–6. https://doi.org/10.1016/j.jort.2016.04.006
63. James, L., Olsen, L. S., & Karlsdóttir, A. (2020). Sustainability and cruise tourism in the arctic: Stakeholder perspectives from Ísafjörður, Iceland and Qaqortoq, Greenland. Journal of Sustainable Tourism, 28(9), 1425–1441. https://doi.org/10.1080/09669582.2020.1745213
64. Jeevan, J., Othman, M. R., Abu Hasan, Z. R., Pham, T. Q. M., & Park, G. K. (2019). Exploring the development of Malaysian seaports as a hub for tourism activities. Maritime Business Review, 4(3), 310–327. https://doi.org/10.1108/MABR-12-2018-0049
65. Jirásek, I., & Hurych, E. (2019). Experience of long-term transoceanic sailing: Cape Horn example. Special Issue on Tourism Research on Adventure Tourism - Current Themes and Developments; Guest Edited by Manuel Sand and Sven Gross., 28, 100221. https://doi.org/10.1016/j.jort.2019.04.003
66. Khodadadi, M. (2018). The emergence of cruise tourism in Iran. Journal of Tourism Futures, 4(3), 275–281. https://doi.org/10.1108/JTF-04-2018-0012
67. Kim, S.-B., Marshall, L. H., Gardiner, R., & Kim, D.-Y. (2021). Conflicts in communities and residents’ attitudes toward the impacts of cruise tourism in the Bahamas. Journal of Travel & Tourism Marketing, 38(9), 956–973. https://doi.org/10.1080/10548408.2021.2006859
68. Knight, D. W., Xiong, L., Lan, W., & Gong, J. (2020). Impact of COVID-19: Research note on tourism and hospitality sectors in the epicenter of Wuhan and Hubei Province, China. International Journal of Contemporary Hospitality Management, 32(12), 3705–3719. https://doi.org/10.1108/IJCHM-04-2020-0278
69. Korbee, D., Mol, A. P. J., & van Tatenhove, J. P. M. (2015). Ecological considerations in constructing marine infrastructure: The Falmouth cruise terminal development, Jamaica. Marine Policy, 56, 23–32. https://doi.org/10.1016/j.marpol.2015.02.003
70. Kumar, N., Trupp, A., & Pratt, S. (2022). Linking tourists’ and micro-entrepreneurs’ perceptions of souvenirs: The case of Fiji. Asia Pacific Journal of Tourism Research, 27(1), 1–14. https://doi.org/10.1080/10941665.2021.1998160
71. Jordan, L.-A. (2013). A critical assessment of Trinidad and Tobago as a cruise homeport: Doorway to the South American cruise market? Maritime Policy & Management, 40(4), 367–383. https://doi.org/10.1080/03088839.2013.777980
72. Lasso, A., & Dahles, H. (2018). Are tourism livelihoods sustainable? Tourism development and economic transformation on Komodo Island, Indonesia. Asia Pacific Journal of Tourism Research, 23(5), 473–485. https://doi.org/10.1080/10941665.2018.1467939
73. Lau, Y., & Yip, T. L. (2020). The Asia cruise tourism industry: Current trend and future outlook. The Asian Journal of Shipping and Logistics, 36(4), 202–213. https://doi.org/10.1016/j.ajsl.2020.03.003
74. Lester, S. E., White, C., Mayall, K., & Walter, R. K. (2016). Environmental and economic implications of alternative cruise ship pathways in Bermuda. Ocean & Coastal Management, 132, 70–79. https://doi.org/10.1016/j.ocecoaman.2016.08.015
75. Lin, L.-P. (Lynn), Yu, C.-Y., & Chang, F.-C. (2018). Determinants of CSER practices for reducing greenhouse gas emissions: From the perspectives of administrative managers in tour operators. Tourism Management, 64, 1–12. https://doi.org/10.1016/j.tourman.2017.07.013
76. Lloret, J., Carreño, A., Carić, H., San, J., & Fleming, L. E. (2021). Environmental and human health impacts of cruise tourism: A review. Marine Pollution Bulletin, 173, 112979. https://doi.org/10.1016/j.marpolbul.2021.112979
77. London, W. R., Lohmann, G., & Moyle, B. D. (2021). Network fragmentation and risk in cruise tourism infrastructure development: Auckland, New Zealand. Case Studies on Transport Policy, 9(1), 336–347. https://doi.org/10.1016/j.cstp.2021.01.011
78. London, W. R., & Lohmann, G. (2014). Power in the context of cruise destination stakeholders’ interrelationships. Cruises and Cruise Ports: Structures and Strategies, 13, 24–35. https://doi.org/10.1016/j.rtbm.2014.11.004
79. Lopes, M. J., & Dredge, D. (2018). Cruise Tourism Shore Excursions: Value for Destinations? Tourism Planning & Development, 15(6), 633–652. https://doi.org/10.1080/21568316.2017.1366358
80. López-Marfil, L., Fernández-Gámez, M. A., Campos-Soria, J. A., & Alaminos, D. (2021). Passengers’ behavioural intentions towards cruise port of call: Evidence from senior tourists. Anatolia, 32(4), 628–642. https://doi.org/10.1080/13032917.2021.1999759
81. MacNeill, T., & Wozniak, D. (2018). The economic, social, and environmental impacts of cruise tourism. Tourism Management, 66, 387–404. https://doi.org/10.1016/j.tourman.2017.11.002
82. Marsh, E. A. (2012). The effects of cruise ship tourism in coastal heritage cities. Journal of Cultural Heritage Management and Sustainable Development, 2(2), 190–199. https://doi.org/10.1108/20441261211273662
83. McCaughey, R., Mao, I., & Dowling, R. (2018). Residents’ perceptions towards cruise tourism development: The case of Esperance, Western Australia. Tourism Recreation Research, 43(3), 403–408. https://doi.org/10.1080/02508281.2018.1464098
84. McFarlane-Morris, S. (2021). ‘Come this close, but no closer!’ Enclave tourism development and social change in Falmouth, Jamaica. Journal of Tourism and Cultural Change, 19(1), 132–146. https://doi.org/10.1080/14766825.2019.1676253
85. McLean, E. G. (2022). Can Jamaica’s cruise administrators re-strategize, strengthen inclusiveness, expand attractions and gather more revenue? Worldwide Hospitality and Tourism Themes, 14(2), 156–161. https://doi.org/10.1108/WHATT-11-2021-0150
86. Meyer-Arendt, K. J., & Lew, A. A. (2013). New Perspectives on Tropical Coastal and Island Tourism Development. Tourism Geographies, 15(1), 1–2. https://doi.org/10.1080/14616688.2012.727100
87. Muñoz Barriga, A. (2017). Percepciones de la gestión del turismo en dos reservas de biosfera ecuatorianas: Galápagos y Sumaco. Investigaciones Geográficas, Boletín Del Instituto de Geografía, 2017(93), 110–125. https://doi.org/10.14350/rig.47805
88. Muritala, B. A., Hernández-Lara, A.-B., Sánchez-Rebull, M.-V., & Perera-Lluna, A. (2022). #CoronavirusCruise: Impact and implications of the COVID-19 outbreaks on the perception of cruise tourism. Tourism Management Perspectives, 41, 100948. https://doi.org/10.1016/j.tmp.2022.100948
89. Nagel, T. W. S. (2020). Northwest Passages: Framing by Local, National and Public Media in Canada’s Arctic. Journalism Practice, 14(10), 1244–1263. https://doi.org/10.1080/17512786.2019.1693280
90. Navarro-Ruiz, S., Casado-Díaz, A. B., & Ivars-Baidal, J. (2020). Cruise tourism: The role of shore excursions in the overcrowding of cities. International Journal of Tourism Cities, 6(1), 197–214. https://doi.org/10.1108/IJTC-04-2018-0029
91. Niavis, S., & Vaggelas, G. (2016). An empirical model for assessing the effect of ports’ and hinterlands’ characteristics on homeports’ potential. Maritime Business Review, 1(3), 186–207. https://doi.org/10.1108/MABR-06-2016-0013
92. Nikčević, J. (2019). Strengthening the role of local government to ensure sustainable development of the cruise sector: The case of Kotor. Marine Policy, 109, 103693. https://doi.org/10.1016/j.marpol.2019.103693
93. Pagoni, I., & Psaraki, V. (2014). A tool for calculating aircraft emissions and its application to Greek airspace. Transportation Planning and Technology, 37(2), 138–153. https://doi.org/10.1080/03081060.2013.851510
94. Paiano, A., Crovella, T., & Lagioia, G. (2020). Managing sustainable practices in cruise tourism: The assessment of carbon footprint and waste of water and beverage packaging. Tourism Management, 77, 104016. https://doi.org/10.1016/j.tourman.2019.104016
95. Panzer-Krause, S. (2020). The lost rural idyll? Tourists’ attitudes towards sustainability and their influence on the production of rural space at a rural tourism hotspot in Northern Ireland. Journal of Rural Studies, 80, 235–243. https://doi.org/10.1016/j.jrurstud.2020.09.013
96. Paoli, C., Vassallo, P., Dapueto, G., Fanciulli, G., Massa, F., Venturini, S., & Povero, P. (2017). The economic revenues and the emergy costs of cruise tourism. Journal of Cleaner Production, 166, 1462–1478. https://doi.org/10.1016/j.jclepro.2017.08.130
97. Papaefthimiou, S., Maragkogianni, A., & Andriosopoulos, K. (2016). Evaluation of cruise ships emissions in the Mediterranean basin: The case of Greek ports. International Journal of Sustainable Transportation, 10(10), 985–994. https://doi.org/10.1080/15568318.2016.1185484
98. Papaefthimiou, S., Maragkogianni, A., & Andriosopoulos, K. (2016). Evaluation of cruise ships emissions in the Mediterranean basin: The case of Greek ports. International Journal of Sustainable Transportation, 10(10), 985–994. https://doi.org/10.1080/15568318.2016.1185484
99. Papathanassis, A. (2017). Cruise tourism management: State of the art. Tourism Review, 72(1), 104–119. https://doi.org/10.1108/TR-01-2017-0003
100. Papathanassis, A. (2020). The growth and development of the cruise sector: A perspective article. Tourism Review, 75(1), 130–135. https://doi.org/10.1108/TR-02-2019-0037
101. Papathanassis, A. (2020). Current issues in cruise tourism: Deconstructing the 6th International Cruise Conference. Current Issues in Tourism, 23(14), 1711–1717. https://doi.org/10.1080/13683500.2019.1654984
102. Parola, F., Satta, G., Penco, L., & Persico, L. (2014). Destination satisfaction and cruiser behaviour: The moderating effect of excursion package. Cruises and Cruise Ports: Structures and Strategies, 13, 53–64. https://doi.org/10.1016/j.rtbm.2014.11.001
103. Pashkevich, A., & Stjernström, O. (2014). Making Russian Arctic accessible for tourists: Analysis of the institutional barriers. Polar Geography, 37(2), 137–156. https://doi.org/10.1080/1088937X.2014.919040
104. Penco, L., & Di Vaio, A. (2014). Monetary and non-monetary value creation in cruise port destinations: An empirical assessment. Maritime Policy & Management, 41(5), 501–513. https://doi.org/10.1080/03088839.2014.930934
105. Pesce, M., Terzi, S., Al-Jawasreh, R. I. M., Bommarito, C., Calgaro, L., Fogarin, S., Russo, E., Marcomini, A., & Linkov, I. (2018). Selecting sustainable alternatives for cruise ships in Venice using multi-criteria decision analysis. Science of The Total Environment, 642, 668–678. https://doi.org/10.1016/j.scitotenv.2018.05.372
106. Pinnock, F., Ajagunna, I., & Casanova, S. (2017). Analysis of market conditions for logistics services and tourism: A Caribbean perspective. Worldwide Hospitality and Tourism Themes, 9(1), 4–16. https://doi.org/10.1108/WHATT-11-2016-0065
107. Pinnock, F. (2014). The future of tourism in an emerging economy: The reality of the cruise industry in Caribbean. Worldwide Hospitality and Tourism Themes, 6(2), 127–137. https://doi.org/10.1108/WHATT-12-2013-0052
108. Pisapia, C., El Kateb, A., Hallock, P., & Spezzaferri, S. (2017). Assessing coral reef health in the North Ari Atoll (Maldives) using the FoRAM Index. Marine Micropaleontology, 133, 50–57. https://doi.org/10.1016/j.marmicro.2017.06.001
109. Polat, N. (2015). Technical Innovations in Cruise Tourism and Results of Sustainability. World Conference on Technology, Innovation and Entrepreneurship, 195, 438–445. https://doi.org/10.1016/j.sbspro.2015.06.486
110. Pounder, P. (2021). Responsible leadership and COVID-19: Small Island making big waves in cruise tourism. International Journal of Public Leadership, 17(1), 118–131. https://doi.org/10.1108/IJPL-08-2020-0085
111. Pranić, L., Marušić, Z., & Sever, I. (2013). Cruise passengers’ experiences in coastal destinations – Floating “B&Bs” vs. Floating “resorts”: A case of Croatia. Ocean & Coastal Management, 84, 1–12. https://doi.org/10.1016/j.ocecoaman.2013.07.002
112. Pulina, M., Meleddu, M., & Del Chiappa, G. (2013). Residents’ choice probability and tourism development. Tourism Management Perspectives, 5, 57–67. https://doi.org/10.1016/j.tmp.2012.10.003
113. Puriri, A., & McIntosh, A. (2019). A cultural framework for Māori tourism: Values and processes of a Whānau tourism business development. Journal of the Royal Society of New Zealand, 49(sup1), 89–103. https://doi.org/10.1080/03036758.2019.1656260
114. Ramoa, C. E. de A., Flores, L. C. da S., & Herle, F. B. (2020). Environmental sustainability: A strategic value in guiding cruise industry management. Journal of Hospitality and Tourism Insights, 3(2), 229–251. https://doi.org/10.1108/JHTI-01-2019-0006
115. Ren, C., James, L., Pashkevich, A., & Hoarau-Heemstra, H. (2021). Cruise trouble. A practice-based approach to studying Arctic cruise tourism. Tourism Management Perspectives, 40, 100901. https://doi.org/10.1016/j.tmp.2021.100901
116. Renaud, L. (2020). Reconsidering global mobility – distancing from mass cruise tourism in the aftermath of COVID-19. Tourism Geographies, 22(3), 679–689. https://doi.org/10.1080/14616688.2020.1762116
117. Robinson, D., Newman, S. P., & Stead, S. M. (2019). Community perceptions link environmental decline to reduced support for tourism development in small island states: A case study in the Turks and Caicos Islands. Marine Policy, 108, 103671. https://doi.org/10.1016/j.marpol.2019.103671
118. Robles, L. T., Galvão, C. B., & Pereira, S. R. (2015). Cruise shipping in Brazil: An emergent or established market? Tourism Management Perspectives, 16, 298–305. https://doi.org/10.1016/j.tmp.2015.09.003
119. Römhild-Raviart, J., Weeden, C., Jarvis, N., & Pantelidis, I. (2019). Cruising with(out) a conscience? Sustainable discourse in the blogosphere. Hospitality & Society, 9(1), 31–51. https://doi.org/10.1386/hosp.9.1.31_1
120. Roura, RicardoM. (2012). Being there: Examining the behaviour of Antarctic tourists through their blogs. Polar Research, 31(1), 10905. https://doi.org/10.3402/polar.v31i0.10905
121. Ruiz-Guerra, I., Molina-Moreno, V., Cortés-García, F. J., & Núñez-Cacho, P. (2019). Prediction of the impact on air quality of the cities receiving cruise tourism: The case of the Port of Barcelona. Heliyon, 5(3), e01280. https://doi.org/10.1016/j.heliyon.2019.e01280
122. Sanches, V. L., Aguiar, M. R. da C. M., de Freitas, M. A. V., & Pacheco, E. B. A. V. (2020). Management of cruise ship-generated solid waste: A review. Marine Pollution Bulletin, 151, 110785. https://doi.org/10.1016/j.marpolbul.2019.110785
123. Sanz Blas, S., & Carvajal-Trujillo, E. (2014). Cruise passengers’ experiences in a Mediterranean port of call. The case study of Valencia. Ocean & Coastal Management, 102, 307–316. https://doi.org/10.1016/j.ocecoaman.2014.10.011
124. Sanz-Blas, S., Buzova, D., & Carvajal-Trujillo, E. (2019). Familiarity and visit characteristics as determinants of tourists’ experience at a cruise destination. Tourism Management Perspectives, 30, 1–10. https://doi.org/10.1016/j.tmp.2019.01.005
125. Shoval, N., Kahani, A., De Cantis, S., & Ferrante, M. (2020). Impact of incentives on tourist activity in space-time. Annals of Tourism Research, 80, 102846. https://doi.org/10.1016/j.annals.2019.102846
126. Simonsen, M., Gössling, S., & Walnum, H. J. (2019). Cruise ship emissions in Norwegian waters: A geographical analysis. Journal of Transport Geography, 78, 87–97. https://doi.org/10.1016/j.jtrangeo.2019.05.014
127. Spencer, A. J., & Spencer, D. (2022). Cruising on choppy seas: The revitalization of Jamaica as a cruise destination post COVID-19. Worldwide Hospitality and Tourism Themes, 14(2), 99–114. https://doi.org/10.1108/WHATT-12-2021-0155
128. Storrie, L., Lydersen, C., Andersen, M., Wynn, R. B., & Kovacs, K. M. (2018). Determining the species assemblage and habitat use of cetaceans in the Svalbard Archipelago, based on observations from 2002 to 2014. Polar Research, 37(1), 1463065. https://doi.org/10.1080/17518369.2018.1463065
129. Sugimura, Y. (2020). Public-private partnerships in Japan’s cruise terminal operations. Research in Transportation Business & Management, 100593. https://doi.org/10.1016/j.rtbm.2020.100593
130. Sun, X., Feng, X., & Gauri, D. K. (2014). The cruise industry in China: Efforts, progress and challenges. International Journal of Hospitality Management, 42, 71–84. https://doi.org/10.1016/j.ijhm.2014.05.009
131. Sun, X., Kwortnik, R., Xu, M., Lau, Y., & Ni, R. (2021). Shore excursions of cruise destinations: Product categories, resource allocation, and regional differentiation. Journal of Destination Marketing & Management, 22, 100660. https://doi.org/10.1016/j.jdmm.2021.100660
132. Taylor, A. R., Barðadóttir, Þ., Auffret, S., Bombosch, A., Cusick, A. L., Falk, E., & Lynnes, A. (2020). Arctic expedition cruise tourism and citizen science: A vision for the future of polar tourism. Journal of Tourism Futures, 6(1), 102–111. https://doi.org/10.1108/JTF-06-2019-0051
133. Tin, T., Summerson, R., & Yang, H. R. (2016). Wilderness or pure land: Tourists’ perceptions of Antarctica. The Polar Journal, 6(2), 307–327. https://doi.org/10.1080/2154896X.2016.1252545
134. Tomej, K., & Lund-Durlacher, D. (2020). Research note: River cruise characteristics from a destination management perspective. Journal of Outdoor Recreation and Tourism, 30, 100301. https://doi.org/10.1016/j.jort.2020.100301
135. Toudert, D., & Bringas-Rábago, N. L. (2016). Impact of the destination image on cruise repeater’s experience and intention at the visited port of call. Ocean & Coastal Management, 130, 239–249. https://doi.org/10.1016/j.ocecoaman.2016.06.018
136. Tovar, B., Espino, R., & López-del-Pino, F. (2020). Residents’ perceptions and attitudes towards the cruise tourism impact in gran Canaria. Research in Transportation Business & Management, 100586. https://doi.org/10.1016/j.rtbm.2020.100586
137. Tseng, P.-H., & Yip, T. L. (2021). An evaluation model of cruise ports using fuzzy analytic hierarchy process. Maritime Business Review, 6(1), 22–48. https://doi.org/10.1108/MABR-01-2020-0004
138. Tsiotas, D., Niavis, S., & Sdrolias, L. (2018). Operational and geographical dynamics of ports in the topology of cruise networks: The case of Mediterranean. Journal of Transport Geography, 72, 23–35. https://doi.org/10.1016/j.jtrangeo.2018.08.001
139. Urbanyi-Popiołek, I. (2014). Cruise Industry in the City of Gdynia, the Implications for Sustainable Logistic Services and Spatial Development. Green Cities - Green Logistics for Greener Cities, Szczecin, 19-21 May 2014, 151, 342–350. https://doi.org/10.1016/j.sbspro.2014.10.032
140. Vairo, T., Quagliati, M., Del Giudice, T., Barbucci, A., & Fabiano, B. (2017). From land- to water-use-planning: A consequence based case-study related to cruise ship risk. Risk Analysis and Land Use Planning: Managing Safety on the Short and Long Range, 97, 120–133. https://doi.org/10.1016/j.ssci.2016.03.024
141. van Bets, L. K. J., Lamers, M. A. J., & van Tatenhove, J. P. M. (2017). Governing cruise tourism at Bonaire: A networks and flows approach. Curated Issue: Transport Mobilities, 12(5), 778–793. https://doi.org/10.1080/17450101.2016.1229972
142. van Bets, L. K. J., Lamers, M. A. J., & van Tatenhove, J. P. M. (2017). Governing cruise tourism at Bonaire: A networks and flows approach. Mobilities, 12(5), 778–793. https://doi.org/10.1080/17450101.2016.1229972
143. Van Bets, L. K. J., Lamers, M. A. J., & van Tatenhove, J. P. M. (2017). Collective self-governance in a marine community: Expedition cruise tourism at Svalbard. Journal of Sustainable Tourism, 25(11), 1583–1599. https://doi.org/10.1080/09669582.2017.1291653
144. van Beukering, P., Sarkis, S., van der Putten, L., & Papyrakis, E. (2015). Bermuda׳s balancing act: The economic dependence of cruise and air tourism on healthy coral reefs. Marine Economics and Policy Related to Ecosystem Services: Lessons from the World’s Regional Seas, 11, 76–86. https://doi.org/10.1016/j.ecoser.2014.06.009
145. Vayá, E., Garcia, J. R., Murillo, J., Romaní, J., & Suriñach, J. (2018). Economic impact of cruise activity: The case of Barcelona. Journal of Travel & Tourism Marketing, 35(4), 479–492. https://doi.org/10.1080/10548408.2017.1363683
146. Verbitsky, J. (2015). Antarctic cruise tourism: A taxing issue? The Polar Journal, 5(2), 311–333. https://doi.org/10.1080/2154896X.2015.1092275
147. Vicente-Cera, I., Acevedo-Merino, A., López-Ramírez, J. A., & Nebot, E. (2020). Use of AIS data for the environmental characterization of world cruise ship traffic. International Journal of Sustainable Transportation, 14(6), 465–474. https://doi.org/10.1080/15568318.2019.1575494
148. Vicente-Cera, I., Acevedo-Merino, A., López-Ramírez, J. A., & Nebot, E. (2020). Use of AIS data for the environmental characterization of world cruise ship traffic. International Journal of Sustainable Transportation, 14(6), 465–474. https://doi.org/10.1080/15568318.2019.1575494
149. Viken, A., & Aarsaether, N. (2013). Transforming an Iconic Attraction into a Diversified Destination: The Case of North Cape Tourism. Scandinavian Journal of Hospitality and Tourism, 13(1), 38–54. https://doi.org/10.1080/15022250.2013.771994
150. Vogt, C., Jordan, E., Grewe, N., & Kruger, L. (2016). Collaborative tourism planning and subjective well-being in a small island destination. Small Island Destinations and Subjective Wellbeing, 5(1), 36–43. https://doi.org/10.1016/j.jdmm.2015.11.008
151. Wahnschafft, R., & Wolter, F. (2022). Assessing tourist willingness to pay for excursions on environmentally benign tourist boats: A case study and trend analysis from Berlin, Germany. Research in Transportation Business & Management, 100826. https://doi.org/10.1016/j.rtbm.2022.100826
152. Walker, K., & Moscardo, G. (2014). Encouraging sustainability beyond the tourist experience: Ecotourism, interpretation and values. Journal of Sustainable Tourism, 22(8), 1175–1196. https://doi.org/10.1080/09669582.2014.918134
153. Walker, K., & Moscardo, G. (2016). Moving beyond sense of place to care of place: The role of Indigenous values and interpretation in promoting transformative change in tourists’ place images and personal values. Journal of Sustainable Tourism, 24(8–9), 1243–1261. https://doi.org/10.1080/09669582.2016.1177064
154. Warmouth, J. M., Skibins, J. C., Storie, M., & Schuler, G. (2021). Melting the ice: Arctic tourists’ epiphanies and connections to wildlife. Journal of Ecotourism, 20(1), 89–97. https://doi.org/10.1080/14724049.2020.1849229
155. Weaver, D. B., & Lawton, L. J. (2017). The cruise shorescape as contested tourism space: Evidence from the warm-water pleasure periphery. Tourism Management Perspectives, 24, 117–125. https://doi.org/10.1016/j.tmp.2017.08.003
156. Wondirad, A. (2019). Retracing the past, comprehending the present and contemplating the future of cruise tourism through a meta-analysis of journal publications. Marine Policy, 108, 103618. https://doi.org/10.1016/j.marpol.2019.103618
157. Yuksel, Y., Yuksel, Z. T., Islek, F., Sahin, C., & Ari Guner, H. A. (2021). Spatiotemporal long-term trends of wind and wave climate and extreme characteristics over the Sea of Marmara. Ocean Engineering, 228, 108946. https://doi.org/10.1016/j.oceaneng.2021.108946
158. Zander, K. K., Saeteros, A., Orellana, D., Granda, V. T., Wegner, A., Izurietah, A., & Garnett, S. T. (2016). Determinants of tourist satisfaction with national park guides and facilities in the Galápagos. International Journal of Tourism Sciences, 16(1–2), 60–82. https://doi.org/10.1080/15980634.2016.1212596
159. Abbasian, S., Onn, G., & Arnautovic, D. (2020). Overtourism in Dubrovnik in the eyes of local tourism employees: A qualitative study. Cogent Social Sciences, 6(1), 1775944. https://doi.org/10.1080/23311886.2020.1775944
160. Akrivos, C., Reklitis, P., & Theodoroyiani, M. (2014). Tourism Entrepreneurship and the Adoption of Sustainable Resources. The Case of Evritania Prefecture. 2nd International Conference on Strategic Innovative Marketing, 148, 378–382. <https://doi.org/10.1016/j.sbspro.2014.07.056>
161. Jordan, E. J., & Vogt, C. A. (2017). Residents’ Perceptions of Stress Related to Cruise Tourism Development. Tourism Planning & Development, 14(4), 527–547. https://doi.org/10.1080/21568316.2017.1287123
